# Molnupiravir, Nirmatrelvir/Ritonavir, or Sotrovimab for High-Risk COVID-19 Patients Infected by the Omicron Variant: Hospitalization, Mortality, and Time until Negative Swab Test in Real Life

**DOI:** 10.3390/ph16050721

**Published:** 2023-05-09

**Authors:** Luca Cegolon, Riccardo Pol, Omar Simonetti, Francesca Larese Filon, Roberto Luzzati

**Affiliations:** 1 Department of Medical, Surgical & Health Sciences, University of Trieste, 34147 Trieste, Italy; larese@units.it (F.L.F.); rluzzati@units.it (R.L.); 2 Occupational Medicine Unit, University Health Agency Giuliano-Isontina (ASUGI), 34129 Trieste, Italy; 3 Infectious Disease Unit, University Health Agency Giuliano-Isontina (ASUGI), 34129 Trieste, Italy; riccardopol91@gmail.com (R.P.);

**Keywords:** SARS-CoV-2, COVID-19, high-risk patients, antivirals, monoclonal antibodies, Sotrovimab, Molnupiravir, Nirmatrelvir/Ritonavir, Paxlovid, mortality, hospitalization

## Abstract

**Background**. Several drugs which are easy to administer in outpatient settings have been authorized and endorsed for high-risk COVID-19 patients with mild–moderate disease to prevent hospital admission and death, complementing COVID-19 vaccines. However, the evidence on the efficacy of COVID-19 antivirals during the Omicron wave is scanty or conflicting. **Methods**. This retrospective controlled study investigated the efficacy of Molnupiravir or Nirmatrelvir/Ritonavir (Paxlovid^®^) or Sotrovimab against standard of care (controls) on three different endpoints among 386 high-risk COVID-19 outpatients: hospital admission at 30 days; death at 30 days; and time between COVID-19 diagnosis and first negative swab test result. Multivariable logistic regression was employed to investigate the determinants of hospitalization due to COVID-19-associated pneumonia, whereas time to first negative swab test result was investigated by means of multinomial logistic analysis as well as Cox regression analysis. **Results**. Only 11 patients (overall rate of 2.8%) developed severe COVID-19-associated pneumonia requiring admission to hospital: 8 controls (7.2%); 2 patients on Nirmatrelvir/Ritonavir (2.0%); and 1 on Sotrovimab (1.8%). No patient on Molnupiravir was institutionalized. Compared to controls, hospitalization was less likely for patients on Nirmatrelvir/Ritonavir (aOR = 0.16; 95% CI: 0.03; 0.89) or Molnupiravir (omitted estimate); drug efficacy was 84% for Nirmatrelvir/Ritonavir against 100% for Molnupiravir. Only two patients died of COVID-19 (rate of 0.5%), both were controls, one (a woman aged 96 years) was unvaccinated and the other (a woman aged 72 years) had adequate vaccination status. At Cox regression analysis, the negativization rate was significantly higher in patients treated with both antivirals—Nirmatrelvir/Ritonavir (aHR = 1.68; 95% CI: 1.25; 2.26) or Molnupiravir (aHR = 1.45; 95% CI: 1.08; 1.94). However, COVID-19 vaccination with three (aHR = 2.03; 95% CI: 1.51; 2.73) or four (aHR = 2.48; 95% CI: 1.32; 4.68) doses had a slightly stronger effect size on viral clearance. In contrast, the negativization rate reduced significantly in patients who were immune-depressed (aHR = 0.70; 95% CI: 0.52; 0.93) or those with a Charlson index ≥5 (aHR = 0.63; 0.41; 0.95) or those who had started the respective treatment course 3+ days after COVID-19 diagnosis (aOR = 0.56; 95% CI: 0.38; 0.82). Likewise, at internal analysis (excluding patients on standard of care), patients on Molnupiravir (aHR = 1.74; 95% CI: 1.21; 2.50) or Nirmatrelvir/Ritonavir (aHR = 1.96; 95% CI: 1.32; 2.93) were more likely to turn negative earlier than those on Sotrovimab (reference category). Nonetheless, three (aHR = 1.91; 95% CI: 1.33; 2.74) or four (aHR = 2.20; 95% CI: 1.06; 4.59) doses of COVID-19 vaccine were again associated with a faster negativization rate. Again, the negativization rate was significantly lower if treatment started 3+ days after COVID-19 diagnosis (aHR = 0.54; 95% CI: 0.32; 0.92). **Conclusions**. Molnupiravir, Nirmatrelvir/Ritonavir, and Sotrovimab were all effective in preventing hospital admission and/or mortality attributable to COVID-19. However, hospitalizations also decreased with higher number of doses of COVID-19 vaccines. Although they are effective against severe disease and mortality, the prescription of COVID-19 antivirals should be carefully scrutinized by double opinion, not only to contain health care costs but also to reduce the risk of generating resistant SARS-CoV-2 strains. Only 64.7% of patients were in fact immunized with 3+ doses of COVID-19 vaccines in the present study. High-risk patients should prioritize COVID-19 vaccination, which is a more cost-effective approach than antivirals against severe SARS-CoV-2 pneumonia. Likewise, although both antivirals, especially Nirmatrelvir/Ritonavir, were more likely than standard of care and Sotrovimab to reduce viral shedding time (VST) in high-risk SARS-CoV-2 patients, vaccination had an independent and stronger effect on viral clearance. However, the effect of antivirals or COVID-19 vaccination on VST should be considered a secondary benefit. Indeed, recommending Nirmatrelvir/Ritonavir in order to control VST in high-risk COVID-19 patients is rather questionable since other cheap, large spectrum and harmless nasal disinfectants such as hypertonic saline solutions are available on the market with proven efficacy in containing VST.

## 1. Background

Several drugs which are easy to administer in outpatient settings—antivirals, human monoclonal antibodies, and immunomodulatory agents—have been authorized and recommended for high-risk COVID-19 patients with mild–moderate disease to prevent hospital admission and death, complementing COVID-19 vaccines [1,2,3,4].

In recent months, these drugs have been widely used worldwide for their supposed proven efficacy in real life [5], since they were endorsed by the respective randomized controlled trials (RCTs) to reduce the risk of hospitalization or death at 28–30 days attributable to COVID-19 in patients with risk factors such as age >50 years, immune depression, or other comorbidities [6,7,8,9,10,11,12,13]. However, most pharmaceutical COVID-19 treatments have been ruled out by the emergence of new variants or are way too expensive or impractical to treat large sectors of the general population [14].

The RCTs of these COVID-19 drugs were conducted on unvaccinated high-risk COVID-19 patients before the Omicron transmission period; hence when more virulent viral strains were circulating [6,7,8,9,10,11]. Omicron, classified as a variant of concern (VOC) on 26 November 2021, rapidly displaced previously circulating strains worldwide, dramatically changing the clinical and therapeutic evidence gathered over the previous two years. Omicron is associated with higher transmission rates, less severe disease, lower hospitalization rates, different clinical presentations and aberrant radiological features [15,16,17,18,19]. Whilst Delta VOC exhibited significant tropism for the ACE2-TMPRSS2 pathway associated with lung infection and increased disease severity, the Omicron BA.1 sub-lineage predominantly targets the upper respiratory tract [20].

Among novel human monoclonal antibodies, Sotrovimab gained attention for its neutralizing efficacy in reducing the risk of progression to severe disease in high-risk COVID-19 patients [21]. However, Sotrovimab needs intravenous (i.v.) administration and monitoring in a health care setting and its efficacy against emerging SARS-CoV-2 variants has been questioned [22,23,24,25].

Two oral antivirals—Molnupiravir and Nirmatrelvir/Ritonavir (Paxlovid^®^)— reportedly more effective than human monoclonal antibodies and easier to administer than Remdesivir, which requires i.v. administration—were authorized between 2021 and 2022 for high-risk COVID-19 patients [9,26]. Molnupiravir is a ribonucleoside prodrug of N-hydroxycytidine (NHC), which forms after oral ingestion of Molnupiravir and circulates systemically to be phosphorylated intracellularly into NHC triphosphate. SARS-CoV-2 polymerase incorporates NHC triphosphate into the viral RNA, which then misguides the latter viral enzyme to incorporate either guanosine or adenosine during viral replication, heaping mutations into the viral genome eventually rendering SARS-CoV-2 unable to replicate [8]. By contrast, Nirmatrelvir/Ritonavir, a novel orally administered antiviral agent manufactured by Pfizer BionNTech, targets SARS-CoV-2 3-chymotrypsin-like cysteine protease enzyme (Mpro), which is essential for the viral replication cycle [7,14].

In addition to being effective and practical to administer, both Molnupiravir and Nirmatrelvir/Ritonavir significantly reduced the baseline viral load and the time between COVID-19 diagnosis and a negative swab test in the respective RCTs [7,8]. A recent observational multi-centric study conducted in France between 24 January 2022 and 5 May 2022 on 255 high-risk COVID-19 patients, all infected by Omicron BA.1 and BA.2, confirmed the effect of Nirmatrelvir/Ritonavir in reducing time until a negative test result [27]. The latter clearance effect against viral infection was reportedly lower for Molnupiravir in other observational studies [28,29].

Despite being effective against disease progression in the respective phase 3 RCTs and a number of observational studies [8,30], the efficacy of Molnupiravir was reportedly lower than that of Nirmatrelvir/Ritonavir [7,8]. In a prospective multi-centric open-label RCT conducted in the UK between December 2021 and April 2022, there was no evidence that Molnupiravir reduced the risk of hospitalization and death attributable to COVID-19 in high-risk patients [12]. The latter study was carried out on 26,411 community COVID-19 patients, largely vaccinated and aged > 50 years and/or with co-morbidities, who were randomly assigned to Molnupiravir plus standard of care (N = 12,821), standard of care alone (N = 12,962), or other treatments (N = 628). Vaccination uptake with the booster was 92% in the Molnupiravir group versus 93% in the control group. Hospitalizations or deaths at 28 days were observed in 1% (=105/12,529) of patients on Molnupiravir plus standard of care against 1% (=98/12,525) of those on standard of care, for an adjusted odds ratio of 1.06 (95% Bayesian credible interval 0.81; 1.41) [12].

Another retrospective observational study was conducted in Hong Kong between 26 February 2022 and 26 June 2022—during the Omicron sub-variant BA.2.2 wave—on 1,074,856 high-risk COVID-19 outpatients, less than half of whom fully vaccinated, who were treated with either Molnupiravir (N = 5383) or Nirmatrelvir/Ritonavir (N = 6464) and followed up for a median time of 103 and 99 days, respectively. Both antivirals were effective in reducing mortality and disease progression. However, while institutionalization was less likely in patients on Nirmatrelvir/Ritonavir (aHR = 0.76; 95% CI: 0.67; 0.86), there was no evidence of a difference between the patients on Molnupiravir and the controls (HR = 0.98; 95% CI: 0.89; 1.06) [31]. Nevertheless, patients on Molnupiravir were older and less vaccinated in the latter study [31].

Other observational studies did not find any efficacy difference between Molnupiravir or Nirmatrelvir/Ritonavir in terms of hospitalization or mortality rate [29,31]. A recent metanalysis, including 9 RCTs and 30,472 patients with mild–moderate COVID-19, reported mild evidence of lower rates of hospitalization, viral clearance, time until viral clearance, and time until symptoms resolution with Molnupiravir as compared to a placebo or standard of care [32].

However, the use of Molnupiravir in high-risk COVID-19 patients was downgraded with respect to Nirmatrelvir/Ritonavir, and following concerns about its clinical benefits expressed by the European Medicines Agency (EMA) on 24 February 2023, the Italian Medicines Agency (AIFA) eventually decided to suspend its use on 13 March 2023 [33].

The evidence on the efficacy of pharmaceutical interventions against hospitalization or mortality for COVID-19 in outpatients during the Omicron wave is scanty or conflicting; yet, it is difficult to disentangle the protective effect of early administration of these drugs from the effect of pre-existing humoral immunity or milder circulating viral strains. Population immunity can in fact be achieved through repeated natural infection and/or vaccination, which in high-income countries is mainly limited by vaccine hesitancy [34].

In view of the above, we conducted an observational controlled clinical study, investigating the efficacy of Molnupiravir or Nirmatrelvir/Ritonavir or Sotrovimab in reducing the risk of hospitalization and death and time until viral clearance when administered to high-risk COVID-19 outpatients, adjusting for potential confounders.

## 2. Results

Table 1 presents each treatment (Molnupiravir, Nirmatrelvir/Ritonavir, and Sotrovimab) by explanatory factors, contrasted with standard of care (controls). As can be seen, the distribution or treatments by sex was rather balanced and the mean age of COVID-19 patients was 68.1 ± 16.2 years (median: 72; IQR: 57; 80). Sixty-one percent of patients received a COVID-19 vaccine booster dose, 12.7% were immunized with two doses, and 18.1% were unvaccinated. Overall, 22.5% of patients were either unvaccinated or had received just one dose of vaccine. Patients affected by immune depression accounted for 23.6% (=91/386) of the total, and the percentage of those affected by severe co-morbidities (Charlson index ≥ 5) was 45.6% (=176/386), whereas 35.5% (=137/386) of patients had a moderate Charlson index (3–4), and 18.9% (=73/386) a mild Charlson index (<3).

As can be noted from Table 1, there was no significant difference between each treatment group and controls), with the exception of immune-depression, whose proportion was significantly higher in patients receiving Molnupiravir (27.6%; *p* = 0.025) or Sotrovimab (31.6%; *p* = 0.014) compared to controls (15.3%). However, patients on Molnupiravir (66.2 ± 18.0 years) or Nirmatrelvir/Ritonavir (66.2 ± 15.4 years) were relatively younger than controls (70.9 ± 14.5 years) and those on Sotrovimab (69.8 ± 16.3 years). Furthermore, the proportion of patients with a Charlson index ≥ 5 was significantly higher among patients treated with Sotrovimab (63.2%; *p* = 0.008) than controls (37.8%), and the proportion of patients undertaking 3+ positive swabs until testing negative for SARS-CoV-2 since COVID-19 diagnosis was significantly lower among those on Nirmatrelvir/Ritonavir (6.9%) compared to controls (22.5%, *p* = 0.004). Finally, time between COVID-19 diagnosis and treatment start was significantly longer in patients on Sotrovimab (mean: 2.5 ± 2.4 days; median 2 (2; 3) days).

No adverse effects were reported in relation to any of the three pharmaceutical treatments under investigation.

### 2.1. Hospitalization and Mortality Attributable to COVID-19

Table 2 shows the distribution of variables by study outcomes. As can be seen, only 13 patients were admitted to hospital—8 (7.2%) controls; 3 (2.9%) treated with Nirmatrelvir/Ritonavir; 2 (3.5%) receiving Sotrovimab; and 0 in the Molnupiravir group. The hospitalization rate was more frequent among females (4.4% = 8/183), unvaccinated patients (5.7% = 4/70), those immunized with three doses of COVID-19 vaccines (3.0% = 7/236), patients who were not immune-depressed (3.7% = 11/295) or those with a Charlson index ≥ 5 (4.0% = 7/176).

Table 3 shows the clinical pattern of 13 COVID-19 patients (8 females vs. 5 males) admitted to hospital, as well as the 5 patients (all females) who eventually died. As can be noted, two COVID-19 patients did not develop COVID-19-associated pneumonia, and the reason for their hospitalization was abdominal sepsis in one case and dyspnea requiring oxygen support in the other. The remaining 11 patients (=2.86% = 11/386; 95% CI: 1.59; 5.11%)—mean age 74.1 years, 6 females vs. 5 males—were hospitalized for severe COVID-19-associated pneumonia, but only 2 of them eventually died of COVID-19. Seven out of 11 (=63.6%; 95% CI: 28.8; 88.3%) of the hospitalized COVID-19 patients were controls, 2/11 (=18.2%; 95% CI: 3.5; 58.0%) were treated with Nirmatrelvir/Ritonavir and 2/11 (=18.2%; 95% CI: 3.5; 58.0%) with Sotrovimab. The rate of COVID-19-associated hospitalization in patients treated with Sotrovimab was 1.8% (=1/56), 0 in the Molnupiravir group, and 2.0% (=2/101) with Nirmatrelvir/Ritonavir.

As can be seen in Table 2, only five patients died, four controls (80%) and one (20%) treated with Nirmatrelvir/Ritonavir. Deaths were more frequent in patients aged 80+ years (60% = 3/5), unvaccinated (60% = 3/5), or those who were not immune-depressed (80% = 4/5). All five patients who died had a Charlson index ≥ 5 (Table 2). However, whilst the overall mortality rate was 5/386 (=1.30%; 95% CI: 0.54–3.09%), one patient died outside hospital after being affected by mild–moderate disease, and the cause of death was not attributable to COVID-19. Four out of five patients (all females) eventually died in hospital, but in only two cases COVID-19 was the cause of death, for an overall attributable mortality of 0.5% = 2/386 (95% CI: 0.13; 2.06%). Both of the latter female patients who died of COVID-19 were controls, one (aged 96 years) was unvaccinated, whereas the other (aged 72 years) had been immunized with the booster dose 161 days before being diagnosed with COVID-19 (hence, she could be considered adequately vaccinated, at least according to our criteria).

Overall, 3.1% (=12/384) of the patients were either admitted to hospital or died within the 30 days since COVID-19 diagnosis.

Table 4 shows the distribution of the 11 hospital admissions attributable to severe COVID-19 pneumonia by explanatory factors. As can be seen, 7 patients were controls, two were treated with Nirmatrelvir/Ritonavir, and one with Sotrovimab. No patient treated with Molnupiravir was admitted to hospital. Compared to controls, hospitalization was less likely in patients on Nirmatrelvir/Ritonavir (aOR = 0.16; 95% CI: 0.03; 0.89) or Molnupiravir (omitted estimate, since zero patients of this group developed severe disease requiring institutionalization). This resulted in an efficacy against severe COVID-19 of 84% (1-aOR = 100%-16%) for Nirmatrelvir/Ritonavir versus 100% (1-aOR = 100%-0%) for Molnupiravir. 

Furthermore, zero patients immunized with four doses of COVID-19 vaccine (N = 14) were admitted to hospital for COVID-19-associated pneumonia; yet, the risk of institutionalization also decreased for Sotrovimab treatment or 2–3 doses of COVID-19 vaccine, despite the fact that the numbers involved were too small to achieve enough statistical power. A progressively lower risk of hospital admission with increasing number of days before treatment start likely reflects a higher proportion of controls starting treatment (standard of care) on day 0 (Table 4).

**Figure 1:**
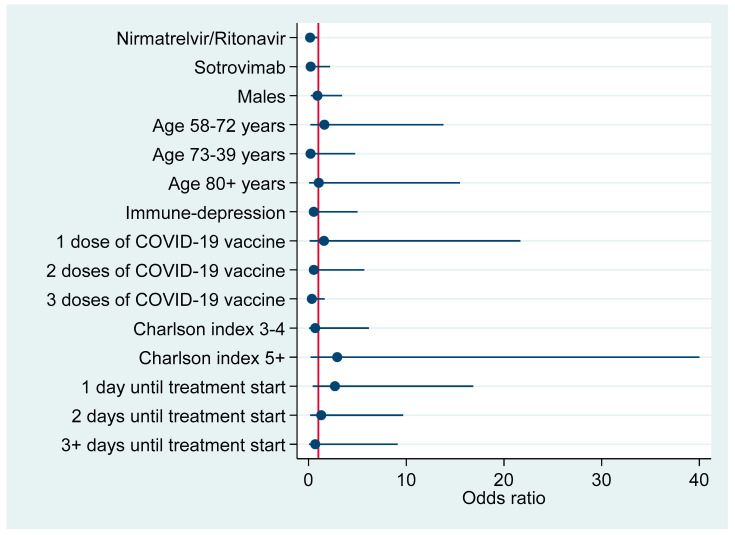
Risk factors for hospitalization due to COVID-19-associated pneumonia (adjusted odds ratios with 95% confidence interval).

Figure 1 plots factors associated with hospitalization due to COVID-19 pneumonia according to results of the above multivariable logistic regression model (Table 4).

### 2.2. Time between COVID-19 Diagnosis and First Negative Swab Test

As can be seen in Table 2, the mean time until first negative swab test was 16.5 ± 10.5 days for Sotrovimab (median = 10; IQR: 14–19), 11.7 ± 5.2 days for Molnupiravir (median = 8; IQR: 11–14), 13.0 ± 7.7 days for controls (median = 11; IQR: 8–15); and 10.2 ± 4.4 days for Nirmatrelvir/Ritonavir (median: 9; IQR: 7–12). Moreover, the positivity window against SARS-CoV-2 increased with age, immune depression, and Charlson index, whereas it diminished with higher number of doses of COVID-19 vaccine received.

Table 5 shows the distribution of negative swab tests by number of days after COVID-19 diagnosis among patients treated with Nirmatrelvir/Ritonavir versus controls. As can be seen, the Absolute Risk Increase (ARI) for Nirmatrelvir/Ritonavir was positive during the first 5–9 days after COVID-19 diagnosis (OR = 2.85; 95% CI: 1.61; 5.06). According to number need to treat (NNT) analysis, four patients (95% CI: 3–8) had to be treated for 9 days to achieve one negative swab test result during the first 5–9 days after COVID-19 diagnosis. However, whilst the respective crude odds ratio (OR) was 2.85 (95% CI: 1.61; 5.06), the corresponding crude hazard ratio (HR =1.01) was non-significant (95% CI: 0.65; 1.57).

Table 6 shows the results of regression analysis (multinomial as well as Cox) for the days since COVID-19 diagnosis until first negative swab test. According to multinomial logistic regression model, patients treated with Nirmatrelvir/Ritonavir were less likely to turn negative for SARS-CoV-2 at 10–14 days (aRRR = 0.21; 95% CI: 0.09; 0.53) or 15+ days (aRRR = 0.19; 95% CI: 0.05; 0.64) than controls. However, COVID-19 vaccination had a stronger effect size than antiviral treatment since the estimates for negativization at 10–14 days were (aRRR = 0.15; 95% CI: 0.06; 0.35) and (aRRR = 0.15; 95% CI: 0.02; 1.00) for three and four doses of COVID-19 vaccine, respectively. Likewise, patients vaccinated with the booster (aRRR = 0.07; 95% CI: 0.02; 0.23) or four doses (aRRR = 0.03; 95% CI: 0.00; 0.45) were also less likely to turn negative at 15+ days after COVID-19 diagnosis.

According to multivariable Cox regression analysis in the entire cohort (Table 6) the negativization rate was significantly higher in patients treated with both antivirals—Nirmatrelvir/Ritonavir (aHR = 1.68; 95% CI: 1.25; 2.26) or Molnupiravir (aHR = 1.45; 95% CI: 1.08; 1.94). However, COVID-19 vaccination with three (aHR = 2.03; 95% CI: 1.51; 2.73) or four (aHR = 2.48; 95% CI: 1.32; 4.68) doses was also associated with earlier negativization, with even stronger effect sizes. By contrast, the rate of negativization diminished significantly in patients immune-depressed (aHR = 0.70; 95% CI: 0.52; 0.93) or those with a Charlson index ≥5 (aHR = 0.63; 0.41; 0.95) or those starting the respective treatment course 3+ days after COVID-19 diagnosis (aOR = 0.56; 95% CI: 0.38; 0.82). Restricting the analysis to the first 5–9 days after COVID-19 diagnosis, the effect on negativization vanished for both antivirals, whereas it was confirmed for any number of doses of COVID-19 vaccine, particularly four (aHR = 5.31; 95% CI: 1.82; 15.51). Among patients turning negative at 15+ days after COVID-19 diagnosis, the rate of negativization was significantly lower in those who were immune-depressed (aHR = 0.52; 95% CI: 0.27; 0.97).

**Figure 2:**
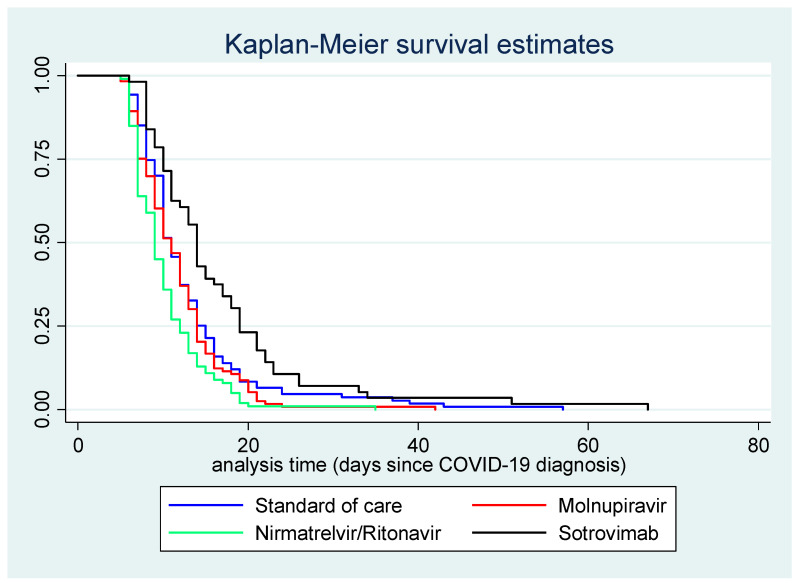
Kaplan–Meier survival curve for time (in days) between COVID-19 diagnosis and first negative swab test, by treatment.

Figure 2 displays the Kaplan–Meier survival curve for time (in days) between COVID-19 diagnosis and first negative swab test, by treatment course.

**Figure 3:**
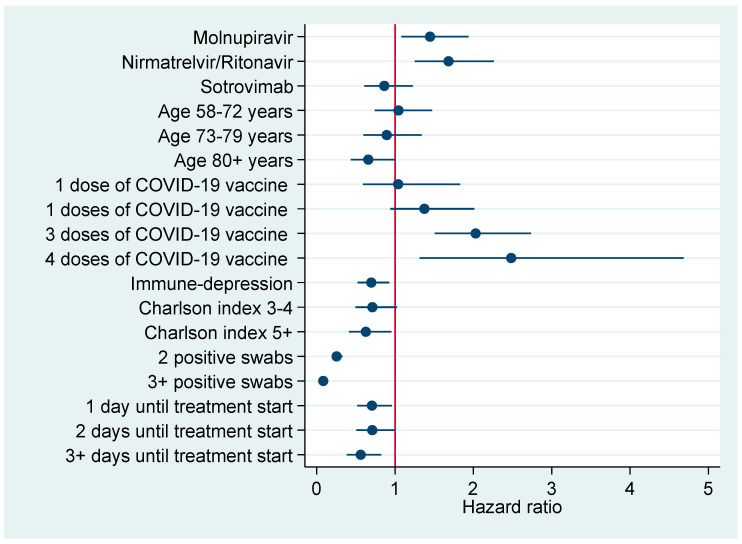
Factors associated with earlier negativization for COVID-19 in the entire cohort of patients (N = 386), reporting adjusted hazard ratios with 95% confidence interval.

**Figure 4:**
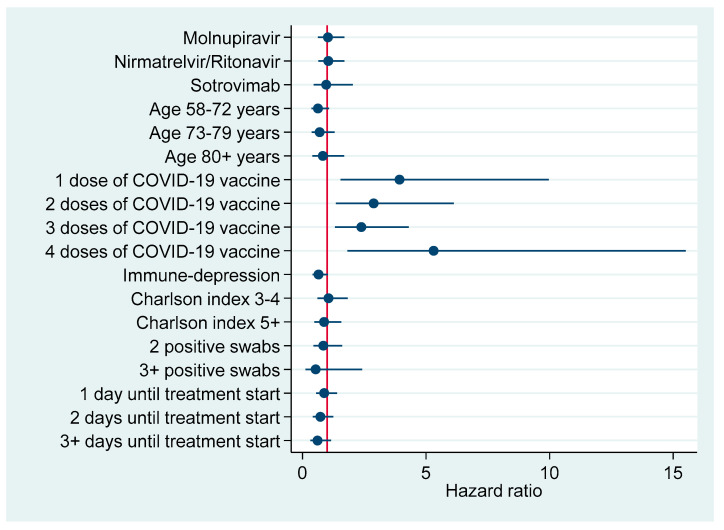
Factors associated with earlier negativization for COVID-19 among patients turning negative during the first 5–9 days after COVID-19 diagnosis (N = 144), reporting adjusted hazard ratios with 95% confidence interval.

Figure 3 and Figure 4 plot factors associated with earlier negativization for SARS-CoV-2 based upon results of multivariable Cox regression analysis (Table 6), reporting an adjusted HR with a 95% CI. Figure 3 refers to the entire cohort (N=386), whereas in Figure 4 the analysis was restricted to patients turning negative in the first 5-9 days since COVID-19 diagnosis.

Table 7 shows a Cox regression model excluding patients on standard of care; it compares the negativization rate for SARS-CoV-2 of both antivirals (Molnupiravir or Nirmatrelvir/Ritonavir) against Sotrovimab (reference category) adjusting for the same explanatory factors displayed in Table 6. Whilst Model 1 was fitted to the entire cohort, Model 2 was restricted to patients turning negative during the first 5–9 days after COVID-19 diagnosis. As can be seen in Model 1, patients on Molnupiravir (aHR = 1.74; 95% CI: 1.21; 2.50) or Nirmatrelvir/Ritonavir (aHR = 1.96; 95% CI: 1.32; 2.93) were more likely to become negative earlier than those on Sotrovimab. However, immunization with three (aHR = 1.91; 95% CI: 1.33; 2.74) or four (aHR = 2.20; 95% CI: 1.06; 4.59) doses of COVID-19 vaccine had a slightly stronger effect size. By contrast, factors associated with a lower negativization rate included treatment delayed 3+ days after COVID-19 diagnosis (aHR = 0.54; 95% CI: 0.32; 0.92), age of 80+ years (aHR = 0.60; 95% CI: 0.37; 0.97) or immune depression (aHR = 0.68; 95% CI: 0.49; 0.95).

In Model 2, restricting the analysis to patients turning negative 5–9 days after COVID-19 diagnosis, the effect of the two antivirals vanished, whereas the vaccination’s effect was reinforced for any dose number, particularly four (aHR = 5.55; 95% CI: 1.58; 19.45), although the estimates for the booster did not reach statistical significance (Table 7).

## 3. Discussion

### 3.1. Main Findings

In this retrospective real-world clinical study on 386 high-risk patients infected by the Omicron variant (mainly BA.1 and BA.2 like), only 11 cases (rate of 2.8%) of severe COVID-19-associated pneumonia requiring admission to hospital were observed. The mean age of the latter 11 institutionalized patients was 74.1 years. Eight (7.2%) of the latter 11 patients were controls, two (1.8%) were treated with Sotrovimab, and two (2.0%) were on Nirmatrelvir/Ritonavir. No patient on Molnupiravir was admitted to hospital. Therefore, patients receiving any of the above three treatment courses exhibited fewer hospitalizations for COVID-19-associated pneumonia with respect to controls; however, due to small numbers involved in the Sotrovimab group, the estimates were significant only for Molnupiravir (100% efficacy) or Nirmatrelvir/Ritonavir (84% efficacy). No adverse effects were reported in relation to any of the three pharmaceutical COVID-19 treatments under investigation.

Only five patients died (rate of 1.3%), but in only two cases death was attributable to COVID-19 (rate of 0.5%). The latter two patients were both controls, females, one 96 years old (unvaccinated for COVID-19) and the other was 72 years old (immunized with the booster dose 161 days before infection; hence, her vaccination status was considered adequate).

In the entire cohort, time between COVID-19 diagnosis and first negative test result ranged between 5 and 67 days (without the exclusion of any potential outlier), with a mean of 12.4 days and a median of 11 days. The median time until first negative swab test was 10 days for patients on Sotrovimab, 8 days for those on Molnupiravir, 7 days for those on Nirmatrelvir/Ritonavir, and 11 days for controls.

Patients on Nirmatrelvir/Ritonavir were more likely than patients on standard of care to turn negative during the first 5–9 days after COVID-19 diagnosis. In particular, according to NNT analysis, four patients had to be treated with Nirmatrelvir/Ritonavir to achieve one negative swab test result within the first 5–9 days after COVID-19 diagnosis. Multinomial logistic regression analysis as well as Cox regression analysis in the entire cohort confirmed higher negativization rates for patients on Nirmatrelvir/Ritonavir. However, COVID-19 vaccination status, particularly 3+ doses, had a more consistent and stronger effect size on negativization against SARS-CoV-2 infection.

At internal Cox regression analysis (excluding patients on standard of care), higher negativization was observed in patients on either antivirals compared to those on Sotrovimab. Nonetheless, 3+ doses of COVID-19 vaccines again exhibited a slightly stronger effect size than the two antivirals. Treatment starting at 3+ days after COVID-19 diagnosis, immune depression and 80+ years of age confirmed to be risk factors for longer viral shedding time (VST). Focusing on patients turning negative for SARS-CoV-2 during the first 5–9 days since COVID-19 diagnosis, the effect of both antivirals vanished, whereas COVID-19 vaccination’s reinforced, particularly four vaccine doses.

### 3.2. Interpretation of Findings

#### 3.2.1. Hospitalization and Mortality Attributable to COVID-19

In the entire cohort, the rate of hospitalizations due to COVID-19 was 2.8% (1.8% in the Sotrovimab group; 2.0% in patients on Nirmatrelvir/Ritonavir; 0% with Molnupiravir; and 7.2 in patients on standard of care), and the death rate was 0.5% (two cases, both controls). These figures were similar to those of other real-world studies carried out during the Omicron transmission period [14,31,35,36,37,38]. Moreover, only two deaths attributable to COVID-19 (rate of 0.5%) were observed; both were patients on standard of care (controls). Molnupiravir, Nirmatrelvir/Ritonavir, and Sotrovimab were therefore all effective in preventing hospital admission and/or mortality due to COVID-19.

Although the numbers involved were too small to draw significant estimates, COVID-19 vaccination (2+ doses) was also effective in preventing institutionalization for COVID-19-associated pneumonia in the present study. Indeed, none of the 14 patients vaccinated with four doses were hospitalized. Moreover, the institutionalization rate was 2.1% in patients immunized with booster against 5.7% in those unvaccinated.

In an observational study conducted in Israel on 180,351 high-risk COVID-19 patients diagnosed between 1 January 2022 and 28 February 2022 (75.1% adequately vaccinated for COVID-19), though Nirmatrelvir/Ritonavir was associated with a lower rate of hospitalization or death at 28 days (HR = 0.54; 95% CI: 0.39; 0.75), adequate COVID-19 vaccination status had a stronger effect size than antiviral treatment on severe COVID-19 or associated mortality (HR = 0.20; 95% CI: 0.17; 0.22]) [14]. In line with other studies, the authors of the latter investigation concluded that COVID-19 vaccination had proven to be the most cost-effective approach to protect vulnerable patients from hospital admission and death due to COVID-19 [14].

In the present study, the efficacy estimates of the three pharmaceutical treatments under investigation were fairly in line with the respective phase 3 RCTs, although latter experimental studies were conducted on unvaccinated high-risk COVID-19 patients before the Omicron transmission period, when more virulent SARS-CoV-2 variants were circulating [6,7,8].

The evidence on the comparative efficacy between the three treatments was rather conflicting in some subsequent observational studies, apart from the already-mentioned UK prospective open-label RCTs on 26,411 high-risk COVID-19 patients assessing the efficacy of Molnupiravir between December 2021 and April 2022 [12] and the large Hong Kong study on 1,074,856 COVID-19 outpatients receiving either Molnupiravir (N = 5383) or Nirmatrelvir/Ritonavir (N = 6464) between 26 February 2022 and 26 June 2022 [31].

For instance, a cohort study was conducted in England on 6020 high-risk community COVID-19 patients with 88% vaccine coverage, receiving either Sotrovimab (N = 3331) or Molnupiravir (N = 2689) between 16 December 2021 and 10 February 2022. Eighty-seven (1.4%) patients were hospitalized at 28 days in the latter study, 0.96% (=32/3331) in the Sotrovimab versus 2.05% (=55/2689) in Molnupiravir group. Furthermore, the mortality rate among the latter 87 hospitalized patients was 0.42% (=25/6020) in the entire cohort: 2.19% (=7/3331) patients on Sotrovimab against 0.67% (=18/2689) of those on Molnupiravir [35].

Another retrospective study from Wales (UK) examined 7,013 non-hospitalized adult COVID-19 patients infected between 16 December 2021 and 22 April 2022—4973 controls against 2040 patients treated either with Molnupiravir (N = 3591; 7.6%) or Nirmatrelvir-Ritonavir (N = 602; 29.5%) or Sotrovimab (1079; 52.9%). Although younger, with less co-morbidities and immunized with higher number of doses of COVID-19 vaccine, 0.4% (=8/2040) of the treated against 10.9% (=544/4973) controls were hospitalized or died in the first 28 days. Patients receiving either Molnupiravir, Nirmatrelvir/Ritonavir, or Sotrovimab were less likely to be hospitalized or to die at multivariable analysis (Hazard rate = 35%; 95% CI: 18–49%), without evidence of efficacy difference by treatment type [38].

Despite recent suspension of Molnupiravir by AIFA, no hospitalizations or deaths due to COVID-19 were observed among patients on latter antiviral in the present study. In a recent systematic analysis, including 9 RCTs on 30,472 COVID-19 patients with mild–moderate disease, there was mild evidence that Molnupiravir consistently reduced mortality (RR = 0.43; 95% CI 0.20;0.94), hospital admission (RR = 0.67; 95% CI 0.45; 0.99), and time before symptoms resolution (mean difference = −2.39 days; 95% CI: −3.71; −1.07) compared to placebo or standard of care [32].

#### 3.2.2. Time between COVID-19 Diagnosis and First Negative Swab Test

The present study found an earlier negativization of SARS-CoV-2 infection in patients treated with both antivirals, particularly Nirmatrelvir/Ritonavir. However, earlier negativization was also consistently observed for increasing number of doses of COVID-19 vaccine, particularly 3+ doses. The effect of both antivirals and vaccination on the negativization rate was observed during the first 5–9 days after COVID-19 diagnosis, waning afterwards. The latter interval fairly corresponds to the environmental temporal persistence of SARS-CoV-2 outside cells. Thereafter, the virus present in the nasal cavity and unable to replicate inside mucosal cells protected by antiviral treatment or pre-existing humoral immunity will progressively extinguish, also for the local response mounted by nasal mucosa of immunocompetent patients. However, patients affected by severe comorbidities or immune depression may struggle to clear SARS-CoV-2 even after 15+ days since infection, as found in the present study. A higher prevalence of immune depression and a Charlson score ≥5 for patients on Sotrovimab may at least partly explain a delayed negativization of this group compared to patients on standard of care, not to mention that the former group started the respective treatment course later, after a median time of 2 days after COVID-19 diagnosis.

Detection and quantification of SARS-CoV-2 load in nasopharyngeal swabs by RT-PCR was a secondary endpoint in the phase 3 RCT of Nirmatrelvir/Ritonavir [7]. Nasopharyngeal or nasal swabs were collected on day 1 (baseline) and days 3, 5, 10, and 14 after COVID-19 diagnosis in the latter experimental study [7]. Adjusting for baseline viral load, serology status and geographic region, the viral load at day 5 was significantly lower in patients on Nirmatrelvir/Ritonavir, by an adjusted mean of an additional 0.868 ± 0.105 log10 copies per milliliter (95% CI: –1.074; –0.6615; *p* < 0.001) if treatment started within 3 days after symptoms onset—a decrease in viral load by a factor of 10 compared to the placebo—and 0.695 ± 0.085 log10 copies per milliliter (95% CI: –0.861; –0.530; *p* < 0.001) if treatment was started within 5 days after symptoms onset. The latter results were also confirmed for patients receiving or assigned to receive treatment with human monoclonal antibodies [7].

Patients on Molnupiravir also exhibited a stronger reduction in viral load from the baseline levels at days 3, 5, and 10 compared to the controls in the respective phase 3 RCT [8]. Likewise, in the above systematic analysis on nine RCTs and 90,472 COVID-19 patients with mild–moderate disease, Molnupiravir was associated with shorter time through viral clearance (mean difference = −1.81 days, 95% CI: −3.31; −0.31) as well as increased rate of viral clearance at 7 days (RR = 3.47, 95% CI: 2.43; 4.96) [32].

A multi-centric French study reported a median time of 11.5 days (95% CI: 10.5–13) until negativization in high-risk COVID-19 patients treated with Sotrovimab, against 4 days (95% CI 4–9) in those on Nirmatrelvir/Ritonavir (*p* < 0.001), who exhibited a faster viral decay (*p* < 0.001). Negativization occurred significantly earlier with increasing baseline PCR cycle threshold values (HR = 1.05; 95% CI: 1.01; 1.08, *p* = 0.01) or Nirmatrelvir/Ritonavir treatment (HR = 2.35; 95% CI: 1.56–3.56, *p* < 0.0001) [27]. Although the latter multicentric study also considered baseline viral load as a predictor, the comparisons were made between two different treatments, Sotrovimab or Nirmatrelvir/Ritonavir, whereas in the present study each individual treatment was also contrasted with patients on standard of care as controls.

The difference in the median time until a negative swab test between the above French multi-centric investigation (4 days) and the present study (7 days) may be explained by differences in the two populations, especially with regard to COVID-19 vaccination coverage. The proportion of patients immunized with 3+ doses of COVID-19 vaccines was 80% in the French study against 64.7% in the present investigation, where 18% of the patients were unvaccinated, 35.8% had an inadequate vaccination status, and 12.9% were immunized with just two doses. Considering only patients on Nirmatrelvir/Ritonavir, the latter percentage increased to 87% in the French study against 67.6% in the present study [27].

In a further clinical investigation at the University of Pisa (Tuscany, Central Italy) on 522 high-risk community COVID-19 patients diagnosed between January 2022 and July 2022, those on Nirmatrelvir/Ritonavir (N = 252) were more likely to turn negative within the first 10 days of treatment (aHR = 1.73; 95% CI: 1.25–2.4; *p* < 0.001) compared with those on Remdesivir (reference category, N = 196), whereas the evidence for patients on Molnupiravir (N = 114) lacked statistical significance (aHR = 1.28; 95% CI: 0.86–1.9; *p* = 0.227) [29]. Again, patients on Nirmatrelvir had higher adequate COVID-19 vaccination status (86.9%) than those on Molnupiravir (74.6%) or controls (77%) in the latter Italian study [29].

In a further observational study by the University of Naples (southern Italy) on 257 high-risk COVID-19 patients diagnosed between 18 February 2022 and 30 June 2022, the median time until a negative swab test was 8 days among 146 (56.8%) patients on Nirmatrelvir/Ritonavir against 10 days in 111 (43.2%) patients on Molnupiravir (*p* < 0.01); these estimates are in line with those of the present study [28]. The time until a negative swab test was 8 days among the 247 (96.1%) patients vaccinated with at least two doses versus 11 days in the 10 patients who were unvaccinated or immunized with only one dose (*p* = 0.306). The latter study reported only crude comparisons, though without controlling for potential confounders [28].

Virtually all authorized COVID-19 vaccines provide more protection against death and severe disease than asymptomatic infection or mild/moderate disease [31]. Nevertheless, the present study confirmed a consistent and stronger effect of COVID-19 vaccination in reducing VST compared to both antivirals under investigation. Recommending antivirals such as Nirmatrelvir/Ritonavir to stop VST in high-risk COVID-19 patients seems questionable, not only efficacy-wise but also from a cost-effectiveness perspective, considering the current cost of a Paxlovid^®^ treatment cycle (USD 530) compared to one dose of COVID-19 vaccine [39], albeit the current price of m-RNA COVID-19 vaccines is expected to be raised to USD 130 per single dose in the USA [40].

Furthermore, there is reasonable concern that antivirals may not remain effective with a rapidly evolving virus, thereby increasing the risk of generating an epidemic of drug resistance in the case of their indiscriminate use to control VST in high-risk COVID-19 patients. Nirmatrelvir/Ritonavir targets the SARS-CoV-2 3-chymotrypsin-like cysteine protease enzyme (Mpro), an essential protein conserved across various coronaviruses. The inhibitory effect of Nirmatrelvir/Ritonavir on the replication of various coronaviruses in vitro has been considered a barrier against antiviral resistance in newly emerging SARS-CoV-2 strains [7]. However, despite reassurances from the industry [7,36,41], in vitro experiments reported that SARS-CoV-2 resistance to Nirmatrelvir readily develops via multiple pathways [42], and several drug-resistant hot spots warranting close monitoring for possible clinical evidence of Paxlovid^®^ resistance have already been identified [43,44,45].

The issue of resistance also applies to human monoclonal antibodies such as Sotrovimab, which maintains neutralizing activity by targeting a conserved SARS-CoV-2 epitope outside the rapidly evolving domain interacting with the respective ACE-2 receptor [21]. As it is effective against all sarbecoviruses, it has been speculated that Sotrovimab, whose parental form S309 was isolated from a SARS-CoV-1 patient, “*would target a highly conserved epitope that would be functionally retained as SARS-CoV-2 evolves*” [21]. Nevertheless, the potential for a reduced efficacy of Sotrovimab against emerging SARS-CoV-2 variants has not been ruled out either [22,23,24,25,46,47,48]. Furthermore, there is theoretical concern about rapid SARS-CoV-2 sub-lineage evolution in the so-called “*human culture medium*” [49].

Other cheap, large spectrum, harmless agents including hypertonic saline solutions are available on the market as nasal disinfectants to effectively reduce VST in COVID-19 patients [50,51,52,53,54,55,56,57]. Hypertonic saline solutions inhibited SARS-CoV-2 replication in vitro yet stimulate the local nasal mucosa to produce hypochlorous acid (HOCl), the active principle of common bleach, a large spectrum agent recommended by the US Environmental Protection Agency for hand washing and fomites disinfection, irrespective of the emerging SARS-CoV-2 variants [58]. In a recent controlled clinical trial on low-risk COVID-19 patients with mild–moderate disease, daily treatment with hypertonic saline solutions sprayed inside the nasal cavity 3 times/day was the only factor independently associated with reduced VST during the first five days after COVID-19 diagnosis [50]

### 3.3. Strengths and Weaknesses

This observational, controlled real-world study assessed the efficacy of three different treatment courses on three separate endpoints among 386 high-risk COVID-19 patients, adjusting for a number of potential confounders. In contrast, some RCTs or observational studies estimated treatment efficacy of pharmacological COVID-19 treatments using composite outcomes combining hospital admission and mortality at 28–30 days.

A further important strength of this study is that all of the hospitalized patients were individually reviewed to exclude eventual institutionalizations and deaths not attributable to COVID-19.

Although randomization is impractical in real life, the baseline distribution of variables was fairly balanced, with the exception of age (relatively lower for the patients on both antivirals), immune depression (more prevalent in patients on Molnupiravir and Sotrovimab), time until treatment start (longer in Sotrovimab group, as expected considering the inclusion criteria), immune depression and Charlson index ≥ 5 (both more prevalent in patients on Sotrovimab).

One important limitation of this study is that since it was conducted in real life, swab tests could not be performed on a daily basis but according to variable time intervals after COVID-19 diagnosis; hence, patients may have turned negative earlier than observed. This means that our results on time until the negative swab test may have underestimated the true negativization rates.

It can be reasonably argued that a larger number of patients would have enabled some statistical risk estimates (such as for Sotrovimab or number doses of COVID-19 vaccines) to turn significant.

Finally, we did not have information on smoking, which is an additional risk factor for severe COVID-19 [7,59].

## 4. Methods

### 4.1. Ethical Considerations

Ethical approval to conduct this observational clinical study was received by the Regional Ethic Committee of Friuli Venezia Giulia Region (CEUR, No. CEUR 2020-OS-072).

Patient consent was waived because according to Italian privacy law (Legislative Decree 101/2018, D. Lgs. 101/2018) patients’ data routinely collected by the Italian National Health Service (Italian NHS) can be used for scientific purposes within the frame of approved studies/protocols, provided that sensitive information is anonymized.

### 4.2. Study Design

A retrospective clinical study was conducted on 386 high-risk COVID-19 patients diagnosed between 1 February 2022 and 31 May 2022 (Omicron circulation > 90%). Patients were infected mainly by BA.1 and BA.2-like variants and were referred by general practitioners or other specialist consultants to the outpatient infectious disease service of Trieste (North-eastern Italy) due to high risk of developing severe COVID-19. High-risk patients were considered those with comorbidities and/or immune depression.

The above high-risk COVID-19 patients were offered treatment with Molnupiravir, Sotrovimab, or Nirmatrelvir/Ritonavir, according to the following criteria:

- Compliance with oral therapy (Molnupiravir or Nirmatrelvir/Ritonavir) over i.v. therapy (Sotrovimab), in which case human monoclonal therapy was opted out;
- Glomerular filtration rate (GFR) < 30 mL/min, contraindicating both Nirmatrelvir/Ritonavir and Molnupiravir, in which case Sotrovimab was recommended;
- Existing therapeutic plans potentially conflicting with Nirmatrelvir/Ritonavir, in which case Molnupiravir or Sotrovimab was proposed for the patient;
- Patient reluctance to receive any of the above treatment options (Molnupiravir, Nirmatrelvir/Ritonavir or Sotrovimab), in which case the patient contributed to the control group (standard of care);
- Late (>5 or 7 days, respectively, for antivirals and monoclonal antibodies) referral to infectious disease outpatient unit following the onset of symptoms, in which case the patient contributed to the control group.

The final number of high-risk COVID-19 patients was eventually broken down as follows:

- 116 patients treated with Molnupiravir;
- 102 receiving Nirmatrelvir/Ritonavir;
- 57 on Sotrovimab;
- 111 receiving standard of care (controls).

Antivirals and monoclonal antibodies were administered according to EMA indications [60,61,62]:

- **Molnupiravir**: 800 mg (4 oral tablets of 200 mg each) twice a day—12 h apart—for 5 days.
- **Nirmatrelvir/Ritonavir**, comprising two oral principles, Nirmatrelvir (150 mg) and Ritonavir (100 mg). Regular dose: two tablets of Nirmatrelvir plus one tablet of Ritonavir to be taken twice a day (12 h apart) for 5 days, for a total daily dosage of 600 mg of Nirmatrelvir plus 200 mg of Ritonavir. Renal impairment dose: one tablet of Nirmatrelvir plus one tablet of Ritonavir to be taken twice a day (12 h apart) for 5 days, for a total daily dosage of 300 mg Nirmatrelvir plus 200 mg of Ritonavir.
- **Sotrovimab:** single dose of 500 mg i.v., following dilution.

### 4.3. Confounding Factors

In addition to age and sex of patients, information was available on number of doses of COVID-19 vaccine, number of positive swab tests undertaken before the first negative swab result, number of days between COVID-19 diagnosis and treatment start, immune depression and Charlson index.

Immune depression was defined as an immune condition sustained by either cancer, leukemia, lymphoma, AIDS, or immunosuppressive medications.

A binary term (yes vs. no) combining number of doses of COVID-19 vaccines with number of days since last dose received was created to account for adequate COVID-19 vaccination status, as follows:

- Patients were considered inadequately vaccinated if they were
  - fully unvaccinated (0 doses); OR
  - immunized with just with one dose of COVID-19 vaccine; OR
  - immunized with 2+ vaccine doses, but last vaccine dose was received >180 days before COVID-19 diagnosis.
- Patients were considered adequately vaccinated if they were
  - immunized with 2+ vaccine doses; AND
  - last vaccine dose was received <181 days before SARS-CoV-2 infection.

### 4.4. Charlson Index

The Charlson co-morbidity index (CCI) was developed by Dr. Mary Charlson in 1987 to classify co-morbidities increasing mortality risk; it was estimated from a cohort study on 604 subjects in a New York hospital over the course of one month back in 1984. CCI was subsequently used to estimate the risk of death associated with breast cancer in 685 patients treated at Yale New Haven hospital between 1962 and 1969 [63]. In its classical form, CCI considers 16 medical conditions stratified by an indicator ranging from 1 to 6, for a final score ranging from 0 to 33. Each score is assigned according to the severity of the condition, particularly the risk of death in one year. CCI was subsequently updated to also include the patient’s age, thus expanding the final score from 0 to 37 [64].

### 4.5. Statistical Analysis

Three separate endpoints were investigated:

- Hospitalizations attributable to COVID-19;
- Mortality attributable to COVID-19;
- Negativization rate, measured in days from COVID-19 diagnosis (first positive RT-PCR result) to first negative swab test (either RT-PCR or third-generation antigenic test) for SARS-CoV-2.

#### 4.5.1. Hospitalization and Mortality Attributable to COVID-19

The rate of hospital admissions at 30 days due to COVID-19 was reviewed in depth to exclude any institutionalization not attributable to COVID-19. Hospitalizations for COVID-19 were then investigated by multiple logistic regression analysis, reporting odds ratio (aOR) with a 95% confidence interval (95% CI), adjusting for age, sex, number of doses of COVID-19 vaccine, immune depression, Charlson index, and time between COVID-19 diagnosis and treatment start.

As there were only five deaths in total—and only two being attributable to COVID-19—only descriptive analysis was used, reporting frequencies and percentages.

#### 4.5.2. Time between COVID-19 Diagnosis and First Negative Swab Test

The effectiveness of Nirmatrelvir/Ritonavir, which exhibited the shortest time through first negative swab test result was investigated by calculating the following proportions:

- **Rate of events in the experimental arm (EER)** = number of events/number of patients in the experimental arm (Nirmatrelvir/Ritonavir course);
- **Rate of events in the control arm (CER)** = number of events/number of patients in the control arm (standard of care).

Using the above data and given that patients receiving Nirmatrelvir/Ritonavir benefited from an early negativization of SARS-CoV-2 infection, the following measurements of clinical significance were estimated for each consecutive day as well as by time intervals (5–9 vs. 10–14 vs. 15+ days) after COVID-19 diagnosis [65]:

- **Absolute risk increase [(ARI) = (EER − CER)]**, expressing the absolute increase of risk of events in the treated compared to control group, accompanied by a 95% CI confidence interval (95% CI). The sign of ARI is positive when EER > CER and negative otherwise.
- **Number needed to treat [(NNT) = (1/ARI)],** expressing the expected number of patients required to obtain one beneficial outcome event, accompanied by 95% CI.
- **Crude odds ratio (OR) with 95% CI**, for the association between EER/CER and the treatment groups (Nirmatrelvir/Ritonavir vs controls), by time interval between COVID-19 diagnosis and negative swab test result (5–9 days; 10–14 days; 15+ days).
- **Crude hazard ratio (HR) with 95% CI,** for the negativization rate by treatment group (Nirmatrelvir/Ritonavir vs. controls) and time interval (5–9 vs. 10–14 vs. 15+ days) between COVID-19 diagnosis and negative swab test result.

The time between COVID-19 diagnosis and first negative swab test result was further investigated by multinomial logistic regression analysis, contrasting the risk of turning negative at 10–14 or 15+ days after COVID-19 diagnosis compared to 5–9 days (base), adjusting for potential confounders displayed in Table 1. The latter analysis yielded an adjusted relative risk ratio (aRRR) with a 95% CI.

The negativization rate was then investigated by multivariable Cox proportional regression analysis, controlling for the explanatory factors displayed in Table 1 and reporting adjusted hazard ratios (aHR) with a 95% CI. The latter approach was applied to the entire cohort of patients and, again, was also broken down by time interval between COVID-19 diagnosis and first negative swab test (5–9 vs. 10–14 vs. 15+ days).

All of the above multinomial and Cox regression models were adjusted for age, sex, number of doses of COVID-19 vaccine, immune depression, Charlson index, time between COVID-19 diagnosis and treatment start, and number of positive swab tests undertaken until negativization. Due to collinearity issue, adequate COVID-19 vaccination status was dropped in order to retain number of doses of COVID-19 vaccines (a more informative term).

Finally, in order to compare the viral clearance efficacy of both antivirals against Sotrovimab, a multivariable Cox proportional regression model was fitted excluding patients on standard of care, investigating the negativization rate of Molnupiravir and Nirmatrelvir/Ritonavir against Sotrovimab (reference category) and adjusting for the same explanatory factors of previous multivariable regression models. The latter Cox model was fitted on the entire cohort (MODEL 1) and restricted to patients testing negative against SARS-CoV-2 during the first 5–9 days since diagnosis (MODEL 2). The results were expressed as aHR (95% CI).

Missing values were excluded and a complete case analysis was performed.

The analysis was conducted by Stata 16 (Stata Corp, College Station, TX, USA).

## 5. Conclusions

In this real-world clinical study on 386 high-risk COVID-19 patients, only 2.8% developed severe COVID-19-associated pneumonia requiring admission to hospital: 1.8% patients on Sotrovimab, 2.0% of those on Nirmatrelvir/Ritonavir, 0% of those on Molnupiravir and 72.7% of patients without treatment. Therefore, patients receiving any of the three latter pharmaceutical interventions had fewer COVID-19 hospitalizations than patients on standard of care, although the risk estimates were significant only for Molnupiravir (100% efficacy) or Nirmatrelvir/Ritonavir (84% efficacy). No adverse effects were reported in relation to any of the three COVID-19 pharmaceutical treatments under investigation.

Only five patients died (rate of 1.3%), and in only two cases death was attributable to COVID-19 (rate of 0.5%). The latter two patients were both controls, one unvaccinated and one adequately immunized against COVID-19.

Although numbers were small, Molnupiravir, Nirmatrelvir/Ritonavir and Sotrovimab were therefore all more effective than standard of care in preventing hospital admission and/or mortality due to COVID-19. However, hospitalizations also decreased also with number of doses of COVID-19 vaccine received.

The median time between COVID-19 diagnosis and first negative swab test was 10 days for Sotrovimab, 8 days for Molnupiravir, 7 days for Nirmatrelvir/Ritonavir and 11 days for standard of care. Patients on both antivirals, particularly Nirmatrelvir/Ritonavir, were more likely to turn negative earlier than patients on standard of care or those on Sotrovimab.

Although they are effective against severe disease and mortality, prescription of antivirals should be carefully scrutinized by double opinion, not only to contain health care costs but also to reduce the risk of generating resistant SARS-CoV-2 strains. Only 64.7% patients were immunized with 3+ doses of COVID-19 vaccine in the present study. High-risk patients should prioritize COVID-19 vaccination, a more cost-effective approach than antivirals against severe COVID-19 pneumonia. Moreover, recommending Nirmatrelvir/Ritonavir in order to control VST in high-risk COVID-19 patients is rather questionable, since vaccination exhibited a consistent and stronger effect size in reducing the time until negative swab test; furthermore, a reduction in VST should in any case be considered a secondary benefit of either COVID-19 vaccines or antivirals. Other cheap, large spectrum, harmless agents such as hypertonic saline solutions are available on the market as powerful nasal disinfectants to effectively reduce VST.

## Figures and Tables

**Table 1:** Comparison of each treatment (Molnupiravir, Nirmatrelvir/Ritonavir, Sotrovimab) with controls, by explanatory factors. Chi-square *p*-value. M = missing information.

Factors	Strata	Total N (%)	Standard of Care N (%) (N = 111)	Molnupiravir (N = 116)		Nirmatrelvir/Ritonavir (N = 102)		Sotrovimab (N = 57)	
				N (%)	*p*-Value	N (%)	*p*-Value	N (%)	*p*-Value
**Sex**	**Female**	183 (47.4)	55 (49.6)	54 (46.6)	0.651	53 (52.0)	0.725	21 (36.8)	0.117
	**Male**	203 (52.6)	56 (50.4)	62 (53.5)		49 (48.0)		36 (63.2)	
**Age** (years)	**Mean ± SD**	68.1 ± 16.2	70.9 ± 14.5	66.2 ± 18.0	0.032	66.2 ± 15.4	0.022	69.8 ± 16.3	0.658
	**Median (IQR)**	72 (57; 80)	74 (60; 82)	68.5 (54; 81)	0.085	68 (57; 78)	0.018	75 (61; 81)	0.913
	**21–57**	97 (25.1)	22 (19.8)	35 (30.2)	0.087	28 (27.5)	0.052	12 (21.1)	0.888
	**58–71**	110 (28.5)	28 (25.2)	33 (28.5)		37 (36.3)		12 (21.1)	
	**73–79**	80 (20.7)	28 (25.2)	16 (13.8)		19 (18.6)		17 (29.8)	
	**80+**	99 (25.7)	33 (29.7)	32 (27.6)		18 (17.7)		16 (28.1)	
**COVID-19** **vaccine doses** (Number)	**0**	70 (18.1)	26 (23.4)	21 (18.1)	0.380	16 (15.7)	0.673	7 (12.3)	0.281
	**1**	17 (4.4)	4 (3.6)	7 (6.0)		4 (3.9)		2 (3.5)	
	**2**	49 (12.7)	11 (9.9)	17 (14.7)		13 (12.8)		8 (14.0)	
	**3**	236 (61.1)	67 (60.4)	64 (55.2)		65 (63.7)		40 (70.2)	
	**4**	14 (3.6)	3 (2.7)	7 (6.0)		4 (3.9)		0	
**Adequate COVID-19** **Vaccination status** (M: 78)	**No**	60 (35.8)	18 (21.7)	19 (20.9)	0.897	17 (20.2)	0.818	6 (12.0)	0.159
	**Yes**	248 (64.2)	65 (78.3)	72 (79.1)		67 (79.8)		44 (88.0)	
**Immune depression**	**No**	295 (76.4)	94 (84.7)	84 (72.4)	0.025	78 (76.5)	0.129	39 (68.4)	0.014
	**Yes**	91 (23.6)	17 (15.3)	32 (27.6)		24 (23.5)		18 (31.6)	
**Charlson** **index**	**<3**	73 (18.9)	24 (21.6)	23 (19.8)	0.203	19 (18.6)	0.853	7 (12.3)	0.008
	**3–4**	137 (35.5)	45 (40.5)	36 (31.0)		42 (41.2)		14 (24.6)	
	**5+**	176 (45.6)	42 (37.8)	57 (49.1)		41 (40.2)		36 (63.2)	
**Positive swabs** **before first negative test** (number)	**M** ± **SD**	2.9 ± 1.3	2.9 ± 1.4	2.8 ± 1.2	0.597	2.6 ± 0.9	0.030	3.4 ± 1.8	0.045
	**Median (IQR)**	2 (2; 3)	2 (2; 3)	2 (2; 3)	0.983	2 (2; 3)	0.123	2 (3; 4)	0.060
	**1**	200 (51.8)	59 (53.2)	59 (50.9)	0.467	59 (57.8)	0.004	23 (40.4)	0.223
	**2**	114 (29.5)	27 (24.3)	36 (31.0)		36 (35.3)		15 (26.3)	
	**3+**	72 (18.7)	25 (22.5)	21 (18.1)		7 (6.9)		19 (33.3)	
**Days between COVID-19 diagnosis and treatment start**	**M** ± **SD**	1.5 ± 1.3	1.4 ± 1.5	1.4 ± 0.9	0.921	1.2 ± 0.9	0.224	2.5 ± 1.8	<0.001
	**Median (IQR)**	1 (1; 2)	1 (0; 2)	1 (1; 2)	0.175	1 (1; 2)	0.977	2 (2; 3)	<0.001
	**0**	36 (13.1)	37 (33.3)	16 (13.8)	0.001	18 (17.7)	<0.001	2 (3.5)	<0.001
	**1**	115 (41.8)	30 (27.0)	51 (44.0)		54 (52.9)		10 (17.5)	
	**2**	81 (29.5)	24 (21.6)	35 (30.2)		23 (22.6)		23 (40.4)	
	**3+**	43 (15.6)	20 (18.0)	14 (12.1)		7 (6.9)		22 (38.6)	

**Table 2:** Distribution of explanatory factors by endpoint. Number (N); column percentage (%); mean (M) ± standard deviation (SD); median with interquartile range (IQR). Total number of patients = 386. Positivity window = days from COVID-19 diagnosis to first negative swab test result.

**Factors**	**Strata**	**Tot** **N (%)**	**Positivity Window (Days)—**Missing: 10		**Hospital Admission** (at 30 Days)			**Mortality** (at 30 Days)		
			**M ± SD**	**M (IQR)**	**Yes** **N (%)**	**No** **N (%)**	***p*-Value**	**Yes** **N (%)**	**No** **N (%)**	***p*-Value**
**Total**		**Range (5; 67)**	**12.4** **±** **7.1**	**11 (8; 14)**	**13 (3.4)**	**373 (96.6)**		**5 (1.3)**	**381 (98.7)**	
**Treatment**	**Standard of care (controls)**	111 (28.8)	13.0 ± 7.7	11 (8; 15)	8 (7.2)	103 (92.8)	0.028	4 (3.6)	107 (96.4)	0.073
	**Molnupiravir**	116 (30.1)	11.7 ± 5.2	11 (8; 14)	0	116 (100)		0	116 (100)	
	**Nirmatrelvir/Ritonavir**	102 (16.4)	10.2 ± 4.4	9 (7; 12)	3 (2.9)	99 (97.1)		1 (1.0)	101 (99.0)	
	**Sotrovimab**	57 (14.8)	16.5 ± 10.5	14 (10; 19)	2 (3.5)	55 (96.5)		0	57 (100)	
**Sex**	**Female**	183 (47.4)	12.1 ± 5.8	8 (11; 14)	8 (4.4)	175 (95.6)	0.299	5 (2.7)	178 (97.3)	0.018
	**Male**	203 (52.6)	12.6 ± 8.1	8 (10; 14)	5 (2.5)	198 (97.5)		0	203 (100)	
**Age** (years)	**21–57**	97 (25.1)	10.9 ± 5.3	10 (7; 13)	4 (4.1)	93 (95.9)	0.697	0	97 (100)	0.292
	**58–72**	110 (28.5)	11.7 ± 6.4	10 (8; 14)	4 (3.6)	106 (96.4)		1 (0.9)	109 (99.1)	
	**73–79**	80 (20.7)	13.9 ± 9.9	12 (8; 16)	1 (1.3)	79 (98.8)		1 (1.3)	79 (98.8)	
	**80+**	99 (25.7)	13.4 ± 6.4	12 (9.5; 16)	4 (4.0)	95 (96.0)		3 (3.0)	96 (97.0)	
**COVID-19** **vaccine doses** (Number)	**0**	70 (18.1)	12.6 ± 4.4	12 (10; 14)	4 (5.7)	66 (94.3)	0.665	3 (4.3)	67 (95.7)	0.177
	**1**	17 (4.4)	13.7 ± 12.5	10 (6; 14)	1 (5.9)	16 (94.1)		0	17 (100)	
	**2**	49 (12.7)	11.8 ± 5.6	10 (7; 14)	1 (2.0)	48 (98.0)		0	49 (100)	
	**3**	236 (61.1)	12.4 ± 7.6	10 (8; 14)	7 (3.0)	229 (97.0)		2 (0.9)	234 (99.1)	
	**4**	14 (3.6)	11.2 ± 4.7	12 (6.8, 14.3)	0	14 (100)		0	14 (100)	
**Immune depression**	**No**	295 (76.4)	11.7 ± 5.8	10 (8; 14)	11 (3.7)	284 (96.3)	0.479	4 (1.4)	291 (98.6)	0.850
	**Yes**	91 (23.6)	14.5 ± 10.1	12 (9; 16)	2 (2.2)	89 (97.8)		1 (1.1)	90 (98.9)	
**Charlson** **index**	**Mild (1–2)**	73 (18.9)	10.0 ± 4.0	9 (7; 11.5)	2 (2.7)	71 (97.3)	0.829	0	73 (100)	0.049
	**Moderate (3–4)**	137 (35.5)	11.8 ± 5.4	11 (8; 14)	4 (2.9)	133 (97.1)		0	137 (100)	
	**Severe (5+)**	176 (45.6)	13.8 ± 8.8	12 (9; 16)	7 (4.0)	169 (96.0)		5 (2.8)	171 (97.2)	
**Positive swabs** **before first negative test** (number)	**1**	200 (51.8)	8.8 ± 2.6	8 (7; 10)	5 (2.5)	195 (97.5)	0.033	2 (1.00)	198 (99.0)	0.043
	**2**	114 (29.5)	12.9 ± 3.7	12 (11; 14)	2 (1.8)	112 (98.3)		0	114 (100)	
	**3+**	72 (18.7)	21.3 ± 10.8	15 (19; 22)	6 (8.3)	66 (91.7)		3 (4.2)	69 (95.8)	

**Table 3:** Hospitalized COVID-19 patients. M = males; F = females; Yrs = years; NIH = National Institute of Health; GGO = ground glass opacities; HFNC = high flow nasal oxygen; NIV = non-invasive ventilation; CRP = C reactive protein; WBC = white blood cells. NA = Not applicable.

Sex	Age (yrs)	COVID-19 Pneumonia	Chest Radiological Pattern	Treatment Arm	Dose of COVID-19 Vaccines	Respiratory Support	NIH Class	Admission Parameters			Admission Indication	Vital Status at 30 Days	Death Cause
								CRP (mg/L)	WBC (10^3^/uL)	D-Dimer (ng/mL FEU)			
M	46	Yes	CT: GGO, consolidation	Sotrovimab	1	HFNC	Severe	134	7.82	573	COVID-19	Survival	-
F	96	Yes	XR: spread GGO	Standard of care (controls)	0	HFNC	Critical	11.9	7.69	632	COVID-19	Death	COVID-19
F	78	Yes	XR: GGO	Standard of care (controls)	3	Venturi mask	Severe	105	6.96	633	COVID-19	Survival	-
F	72	Yes	CT: GGO + consolidation	Standard of care (controls)	3	HFNC/NIV	Critical	17.7	89.43	632	COVID-19	Death	COVID-19
F	94	Yes	XR: consolidation (left middle lobe)	Standard of care (controls)	0	Venturi mask	Severe	157	10.36	1016	COVID-19	Death	Other
M	48	Yes	CT: GGO (ilum)	Nirmatrelvir/Ritonavir	3	Venturi mask	Severe	251	15.78	1130	COVID-19	Survival	-
F	92	Yes	CT: consolidation	Nirmatrelvir/Ritonavir	0	Venturi mask	Severe	16.2	8.20		COVID-19	Death	Other
M	81	Yes	XR: spread GGO	Standard of care (controls)	3	Venturi mask	Severe	7.4	29.17	12.42	COVID-19	Survival	-
F	57	No	NA	Standard of care (controls)	3	Venturi mask	NA	7.6	8.06	-	Other	Survival	-
F	52	No	NA	Nirmatrelvir/Ritonavir	3	Venturi mask	NA	4	4.49	-	Other	Survival	-
M	72	Yes	XR: GGO	Standard of care (controls)	0	Venturi mask	Severe	123	10.29	4164	COVID-19	Survival	-
M	67	Yes	XR: consolidations	Standard of care (controls)	2	Venturi mask	Severe	72.6	7.05	233	COVID-19	Survival	-
F	70	Yes	CT: consolidations, lung embolism	Standard of care (controls)	3	Venturi mask	Severe	67.4	9.65	3859	COVID-19	Survival	-
		**Non-hospitalized COVID-19 patients deceased**											
F	79	No		Standard of care (controls)	3		Mild/moderate					Death	Other

**Table 4:** COVID-19-associated hospitalization by explanatory factors. Number (N), row percentage (%) adjusted odds ratio (aOR) with 95% confidence interval (95% CI) for the respective multiple logistic regression model. Obs. = complete (cases) analysis observations.

Term	Strata	Hospitalization Due to COVID-19 (Missing Values: 2)			Multiple Logistic Regression
**No** **(N = 373)**	**Yes** **(N = 11)**	**Chi-Square** ***p*-Value**	**aOR (95% CI)** (261 Obs.)
**Treatment group**	**Standard of care (controls)**	103 (92.8)	8 (7.2)	0.009	Reference
	**Molnupiravir**	116 (100)	0		Omitted
	**Nirmatrelvir/Ritonavir**	99 (98.0)	2 (2.0)		0.16 (0.03; 0.89)
	**Sotrovimab**	55 (98.2)	1 (1.8)		0.22 (0.02; 2.20)
**Sex**	**Female**	175 (96.7)	6 (3.3)	0.617	Reference
	**Male**	198 (97.5)	5 (2.5)		0.92 (0.24; 3.43)
**Age** (years)	**21–57**	93 (97.9)	2 (2.1)	0.643	Reference
	**58–72**	106 (96.4)	4 (3.6)		1.62 (0.19; 13.80)
	**73–79**	79 (98.8)	1 (1.3)		0.21 (0.01; 4.77)
	**80+**	95 (96.0)	4 (4.0)		1.05 (0.07; 15.50)
**COVID-19** **Vaccine doses** (number)	**0**	66 (94.3)	4 (5.7)	0.466	Reference
	**1**	16 (94.1)	1 (5.9)		1.58 (0.12; 21.69)
	**2**	48 (98.0)	1 (2.0)		0.54 (0.05; 5.71)
	**3**	229 (97.9)	5 (2.1)		0.34 (0.07; 1.66)
	**4**	14 (100)	0		Omitted
**Immune depression**	**No**	284 (96.6)	10 (3.4)	0.254	Reference
	**Yes**	89 (98.9)	1 (1.1)		0.53 (0.06; 5.02)
**Charlson index**	**1–2**	71 (97.3)	2 (2.7)	0.812	Reference
	**3–4**	133 (97.8)	3 (2.2)		0.69 (0.08; 6.17)
	**5+**	169 (96.6)	6 (3.4)		2.94 (0.22; 40.01)
**Days between COVID-19 diagnosis and** **treatment start**	**0**	71 (97.2)	2 (2.7)	0.904	Reference
	**1**	139 (96.5)	5 (3.5)		2.70 (0.43; 16.86)
	**2**	101 (97.1)	3 (2.9)		1.30 (0.17; 9.68)
	**3+**	62 (98.4)	1 (1.6)		0.68 (0.05; 9.13)

**Table 5:** Number of negative swab test results (=study endpoint) in treatment (102 patients receiving Nirmatrelvir/Ritonavir) versus control group (111 patients receiving standard of care), by number of days after COVID-19 diagnosis. Negativization rate in the experimental arm (experimental event rate; EER) versus control group (control event rate; CER); absolute risk increase (ARI), NNT = number needed to treat; OR = crude odds ratio; HR = crude hazard ratio. M: missing information on date of first negative swab test.

Day	Nirmatrelvir/ Ritonavir (M: 2)	Standard of Care (Controls) (M: 4)	EER		CER		ARI		NNT (95% CI)	Crude OR (95% CI)	Crude HR (95% CI)
			Daily	Pooled	Daily	Pooled	Daily	Pooled (95% CI)			
5	1	1	1.0	55.0	0.9	29.9	0.1	25.0% (12.0; 38.0%)	4 (3; 8)	2.85 (1.61; 5.06)	1.01 (0.65; 1.57)
6	14	5	14.0		4.7		9.3				
7	21	10	21.0		9.3		11.7				
8	5	11	5.0		10.3		−5.3				
9	14	5	14.0		4.7		9.3				
10	9	20	9.0	32.0	18.7	44.9	−9.7	−13.0% (−0.26; 0.02)	NA	0.57 (0.33; 1.01)	0.99 (0.63; 1.55)
11	9	6	9.0		5.6		3.4				
12	4	9	4.0		8.4		−4.4				
13	6	5	6.0		4.7		1.3				
14	4	8	4.0		7.5		−3.5				
15	2	4	2.0	13.0	3.7	24.3	−1.7	−11.0 (−21.5; −0.48)	NA	0.47 (0.23; 0.98)	1.43 (0.72; 2.87)
16	2	6	2.0		5.6		−3.6				
17	1	2	1.0		1.9		−0.9				
18	3	2	3.0		1.9		1.1				
19	3	4	3.0		3.7		−0.7				
20	1	0	1.0		0		1.0				
21+	1	9	1.0		8.4		−7.4				
**Total**	**100**	**107**									

**Table 6:** Multinomial logistic regression analysis for negativization rates; results expressed as adjusted relative risk ratios (aRRR) with 95% confidence interval (95% CI). Multiple Cox regression model on the negativization rate against SARS-CoV-2 by days since COVID-19 diagnosis; results expressed as adjusted hazard ratio (aHR) with 95% confidence interval (95% CI). Obs. = complete case analysis observations. Significantly lower negativization rates are highlighted in orange; significant higher negativization rates are marked in green.

**Factors**	**Strata**	Multinomial Regression * aRRR (95% CI) **(Base Category: 5–9 Days)** (376 Obs.)		**COX REGRESSION** aHR (95% CI) *			
		**Days after COVID-19 Diagnosis**		**Full Cohort** (376 Obs.)	**Days after COVID-19 Diagnosis**		
		**10–14**	**15+**		**5–9 ** (144 Obs.)	**10–14** (145 Obs.)	**15+** (87 Obs.)
**Treatment**	**Standard of care** **(controls)**	Reference	Reference	Reference	Reference	Reference	Reference
	**Molnupiravir**	0.47 (0.20; 1.10)	0.31 (0.09; 1.04)	1.45 (1.08; 1.94)	1.03 (0.62; 1.69)	0.91 (0.54; 1.52)	1.96 (1.00; 3.85)
	**Nirmatrelvir/Ritonavir**	0.21 (0.09; 0.53)	0.19 (0.05; 0.64)	1.68 (1.25; 2.26)	1.04 (0.64; 1.69)	1.09 (0.62; 1.91)	1.30 (0.58; 2.96)
	**Sotrovimab**	0.73 (0.24; 2.19)	1.17 (0.29; 4.80)	0.86 (0.61; 1.23)	0.96 (0.45; 2.04)	0.61 (0.34; 1.10)	1.15 (0.56; 2.36)
**Sex**	**Female**	Reference	Reference	Reference	Reference	Reference	Reference
	**Male**	0.69 (0.36; 1.30)	0.70 (0.29; 1.65)	1.15 (0.93; 1.42)	1.07 (0.74; 1.56)	1.19 (0.84; 1.69)	0.82 (0.49; 1.39)
**Age** (years)	**21–57**	Reference	Reference	Reference	Reference	Reference	Reference
	**58–72**	0.89 (0.32; 2.49)	0.44 (0.11; 1.83)	1.04 (0.74; 1.47)	0.62 (0.36; 1.08)	0.84 (0.43; 1.61)	0.76 (0.34; 1.70)
	**73–79**	0.81 (0.24; 2.81)	1.00 (0.20; 5.13)	0.89 (0.60; 1.34)	0.69 (0.37; 1.30)	0.79 (0.38; 1.67)	1.01 (0.41; 2.46)
	**80+**	2.11 (0.60; 7.44)	2.52 (0.49; 13.12)	0.66 (0.43; 1.00)	0.82 (0.40; 1.69)	0.58 (0.28; 1.20)	1.00 (0.40; 2.51)
**COVID-19** **Vaccine doses** (Number)	**0**	Reference	Reference	Reference	Reference	Reference	Reference
	**1**	0.30 (0.07; 1.32)	0.27 (0.03; 2.66)	1.04 (0.59; 1.83)	3.93 (1.55; 9.97)	1.08 (0.39; 2.99)	0.33 (0.09; 1.21)
	**2**	0.33 (0.11; 1.02)	0.36 (0.08; 1.60)	1.37 (0.94; 2.01)	2.88 (1.36; 6.12)	1.36 (0.73; 2.52)	1.23 (0.50; 3.05)
	**3**	0.15 (0.06; 0.35)	0.07 (0.02; 0.23)	2.03 (1.51; 2.73)	2.38 (1.32; 4.30)	1.32 (0.84; 2.08)	1.24 (0.56; 2.74)
	**4**	0.15 (0.02; 1.00)	0.03 (0.00; 0.45)	2.48 (1.32; 4.68)	5.31 (1.82; 15.51)	0.97 (0.32; 2.96)	3.61 (0.83; 15.66)
**Immune** **depression**	**No**	Reference	Reference	Reference	Reference	Reference	Reference
	**Yes**	0.97 (0.42; 2.27)	1.03 (0.34; 3.17)	0.70 (0.52; 0.93)	0.65 (0.41; 1.03)	0.69 (0.42; 1.12)	0.52 (0.27; 0.97)
**Charlson** **index**	**1–2**	Reference	Reference	Reference	Reference	Reference	Reference
	**3–4**	1.78 (0.60; 5.26)	2.29 (0.49; 10.60)	0.71 (0.49; 1.02)	1.05 (0.60; 1.83)	0.78 (0.39; 1.55)	0.47 (0.17; 1.34)
	**5+**	1.94 (0.58; 6.54)	3.15 (0.57; 17.35)	0.63 (0.41; 0.95)	0.87 (0.48; 1.57)	0.89 (0.40; 1.98)	0.36 (0.11; 1.12)
**Days between COVID-19 diagnosis and** **treatment start**	**0**	Reference	Reference	Reference	Reference	Reference	Reference
	**1**	2.08 (0.82; 5.26)	2.65 (0.72; 9.73)	0.71 (0.52; 0.96)	0.88 (0.55; 1.40)	0.79 (0.42; 1.50)	0.87 (0.42; 1.79)
	**2**	2.02 (0.75; 5.43)	2.21 (0.56; 8.72)	0.71 (0.51; 0.99)	0.72 (0.42; 1.25)	0.83 (0.45; 1.55)	0.64 (0.29; 1.42)
	**3+**	4.86 (1.55; 15.23)	8.23 (1.73; 39.09)	0.56 (0.38; 0.82)	0.61 (0.32; 1.15)	0.84 (0.43; 1.64)	1.10 (0.43; 2.77)

* also adjusted for number of positive swab tests performed between COVID-19 diagnosis and first negative swab test.

**Table 7:** Multiple Cox regression model comparing the negativization rate of Nirmatrelvir/Ritonavir or Molnupiravir against Sotrovimab. Estimates expressed as adjusted hazard ratio (aHR) with 95% confidence interval (95% CI). Model 1 fitted to the entire cohort; Model 2 restricted to patients turning negative for SARS-CoV-2 in the first 5–9 days after COVID-19 diagnosis. Significantly lower negativization rates are highlighted in orange; significant higher negativization rates are marked in green.

Term	Strata	Cox Regression aHR (95% CI)	
Model 1 * (269 Obs.)	Model 2 * (112 Obs.)
**Treatment group**	**Sotrovimab (Controls)**	Reference	Reference
	**Molnupiravir**	1.74 (1.21; 2.50)	1.07 (0.50; 2.28)
	**Nirmatrelvir/Ritonavir**	1.96 (1.32; 2.93)	1.05 (0.49; 2.26)
**Sex**	**Female**	Reference	Reference
	**Male**	1.27 (0.98; 1.64)	1.15 (0.74; 1.78)
**Age** (years)	**21–57**	Reference	Reference
	**58–72**	1.00 (0.66; 1.49)	0.53 (0.27; 1.03)
	**73–79**	0.88 (0.55; 1.41)	0.62 (0.29; 1.32)
	**80+**	0.60 (0.37; 0.97)	0.69 (0.31; 1.57)
**COVID-19** **Vaccine doses** (number)	**0**	Reference	Reference
	**1**	0.98 (0.50; 1.93)	3.36 (1.10; 10.24)
	**2**	1.32 (0.83; 2.10)	2.68 (1.06; 6.78)
	**3**	1.91 (1.33; 2.74)	2.06 (0.97; 4.39)
	**4**	2.20 (1.06; 4.59)	5.55 (1.58; 19.45)
**Immune depression**	**No**	Reference	Reference
	**Yes**	0.68 (0.49; 0.95)	0.64 (0.39; 1.06)
**Charlson index**	**1–2**	Reference	Reference
	**3–4**	0.77 (0.49; 1.21)	1.47 (0.74; 2.92)
	**5+**	0.81 (0.49; 1.34)	1.03 (0.53; 1.98)
**Days between** **COVID-19 diagnosis** **and treatment start**	**0**	Reference	Reference
	**1**	0.72 (0.47; 1.09)	0.88 (0.51; 1.51)
	**2**	0.67 (0.42 1.06)	0.67 (0.34; 1.31)
	**3+**	0.54 (0.32; 0.92)	0.67 (0.30; 1.51)

* also adjusted for number of positive swab tests performed between COVID-19 diagnosis and first negative swab test.

## Data Availability

The datasets generated and analyzed during the current study are not publicly available since they were purposively collected by the authors for the present study, but they may be available from the corresponding author on reasonable request.
